# Retraction: Anti-Ulcerogenic Effect of Methanolic Extracts from *Enicosanthellum pulchrum* (King) Heusden against Ethanol-Induced Acute Gastric Lesion in Animal Models

**DOI:** 10.1371/journal.pone.0294008

**Published:** 2023-11-10

**Authors:** The PLOS ONE Editors

Following the publication of this article [1], concerns were raised regarding reuse of results presented in Figures 7 and 8. Specifically,

- Figure 7B of this study [1] appears similar to Figure 6A of [2, retracted in 3]*, despite being used to represent different experimental conditions.
- The Figure 7G panel of this study [1] appears to (partially) overlap with the following results, despite being used to represent different experimental conditions:
  - Figure 7e of [4, retracted in 5]
  - Figure 4e of [6, corrected in 7]*
  - Fig. 8C of [8]
  - Fig. 4e of [9]
  - Figure 5F of [10]
  - Figure 5 panel G3 of [11, retracted in 12]
- Figure 8A of this study [1] appears similar to the Figure 6 panel G7 of [11, retracted in 12]*, despite being used to represent different experimental conditions.
- Figure 8G of this study [1] appears similar to Figure 7a of [4, retracted in 5]* when rotated, despite being used to represent different experimental conditions.

Given the nature and extent of the issues, the *PLOS ONE* Editors are concerned about the reliability of data management and/or reporting for this study [1].

Following editorial communication regarding these concerns, one of the co-authors requested retraction of the article. The original data underlying this study were not provided for editorial review.

In light of the above concerns, the *PLOS ONE* Editors retract this article.

Some figure panels discussed above appear to report previously published material that are offered under a CC BY license, but the original articles were not attributed in [1]. For these images, the * by the citation, above, marks the oldest publication of the image of which PLOS is aware.

NN, SG, PH, NAM, NMH, and MAA agreed with the retraction. MH responded but expressed neither agreement nor disagreement with the editorial decision. SMS, BK, HO, MF, HK, HT, and HMA either did not respond directly or could not be reached. NN, SG, PH, NAM, and NMH apologize for the issues with the published article. MAA stands by the article’s findings.
